# MRI of Auto-Transplantation of Bone Marrow-Derived Stem-Progenitor Cells for Potential Repair of Injured Arteries

**DOI:** 10.1371/journal.pone.0031137

**Published:** 2012-02-17

**Authors:** Yanfeng Meng, Feng Zhang, Tiffany Blair, Huidong Gu, Hongqing Feng, Jinnan Wang, Chun Yuan, Zhaoqi Zhang, Bensheng Qiu, Xiaoming Yang

**Affiliations:** 1 Image-Guided Bio-Molecular Interventions Section, Department of Radiology, University of Washington School of Medicine, Seattle, Washington, United States of America; 2 Department of Radiology, Beijing Anzhen Hospital, Capital Medical University, Beijing, China; 3 Clinical Sites Research Program, Philips Research North America, Briarcliff Manor, New York, United States of America; 4 Vascular Imaging Lab, Department of Radiology, University of Washington School of Medicine, Seattle, Washington, United States of America; University of Nebraska Medical Center, United States of America

## Abstract

**Background:**

This study was to validate the feasibility of using clinical 3.0T MRI to monitor the migration of autotransplanted bone marrow (BM)-derived stem-progenitor cells (SPC) to the injured arteries of near-human sized swine for potential cell-based arterial repair.

**Methodology:**

The study was divided into two phases. For in vitro evaluation, BM cells were extracted from the iliac crests of 13 domestic pigs and then labeled with a T2 contrast agent, Feridex, and/or a fluorescent tissue marker, PKH26. The viability, the proliferation efficiency and the efficacies of Feridex and/or PKH26 labeling were determined. For in vivo validation, the 13 pigs underwent endovascular balloon-mediated intimal damages of the iliofemoral arteries. The labeled or un-labeled BM cells were autotransplanted back to the same pig from which the BM cells were extracted. Approximately three weeks post-cell transplantation, 3.0T T2-weighted MRI was performed to detect Feridex-created signal voids of the transplanted BM cells in the injured iliofemoral arteries, which was confirmed by subsequent histologic correlation.

**Principal Findings:**

Of the in vitro study, the viability of dual-labeled BM cells was 95–98%. The proliferation efficiencies of dual-labeled BM cells were not significantly different compared to those of non-labeled cells. The efficacies of Feridex- and PKH26 labeling were 90% and 100%, respectively. Of the in vivo study, 3.0T MRI detected the auto-transplanted BM cells migrated to the injured arteries, which was confirmed by histologic examinations.

**Conclusion:**

This study demonstrates the capability of using clinical 3.0T MRI to monitor the auto-transplantation of BM cells that migrate to the injured arteries of large animals, which may provide a useful MRI technique to monitor cell-based arterial repair.

## Introduction

Atherosclerotic cardiovascular disease is still the leading cause of death in developed countries [1]. One of the characteristic features of the disease is its diffuse involvement of arteries across the entire human body and the simultaneous presence of multiple atherosclerotic lesions or plaques. Rupture of plaques is often lethal, causing myocardial infarction or stroke, which are responsible for over 50% of all death around the world.

Surgery-based artery bypass grafting and percutaneous transarterial interventions are currently available methods for reducing the risk of myocardial infarction and stroke from atherosclerotic cardiovascular disease. However, these methods have their drawbacks. Surgical bypass grafting is an invasive approach, while percutaneous transarterial intervention only offers local treatment of limited numbers of atherosclerotic arterial segments. Thus, it is necessary to develop new or alternative methods to treat the multiple and diffused atherosclerotic plaques, stabilize vulnerable plaques, and repair ruptured plaques in their “fresh state”.

Recent studies have shown that injured or ruptured atherosclerotic lesions can recruit circulating bone marrow (BM)-derived stem-progenitor cells (SPCs) that are capable of differentiation into proliferating smooth muscle cells (SMCs) and endothelial cells (ECs)[2]. Another important report is that intravenously transfused BM-SPCs can specifically migrate to atherosclerotic lesions [3]. Based on these observations, the concept of “cell-based arterial repair and plaque stabilization” may emerge. In theory, SPCs can be used to stabilize or repair plaques based on their plaque-homing effect and ability to differentiate into SMCs and ECs, which can secrete extracellular matrix rich in glycosaminoglycans and collagen [4]. All of these elements may contribute to strengthen the fibrous caps of plaques and maintain the integrity of the endothelial layer to prevent additional damage to the arterial wall.

Studies have established the “proofs-of-principal” of using MRI to monitor, in vivo, the trafficking of BM-SPCs to the injured arteries or atherosclerotic plaques for potential repair of injured arteries and vulnerable atherosclerotic lesions [5]–[7]. However, these studies were performed in small animals with allo-transplantation of BM-SPCs from donors, which may increase the risk of immunological rejection, and therefore, limit its clinical application on humans. Autologous cell transplantation is one of strategies to solve this problem. The aim of this study was to validate the feasibility of using clinical 3.0T MRI to monitor the migration of autotransplanted BM-SPCs to the injured arteries of large animals, which might open new avenues for cell-based arterial repair under MRI guidance.

## Results

**Figure 1:**
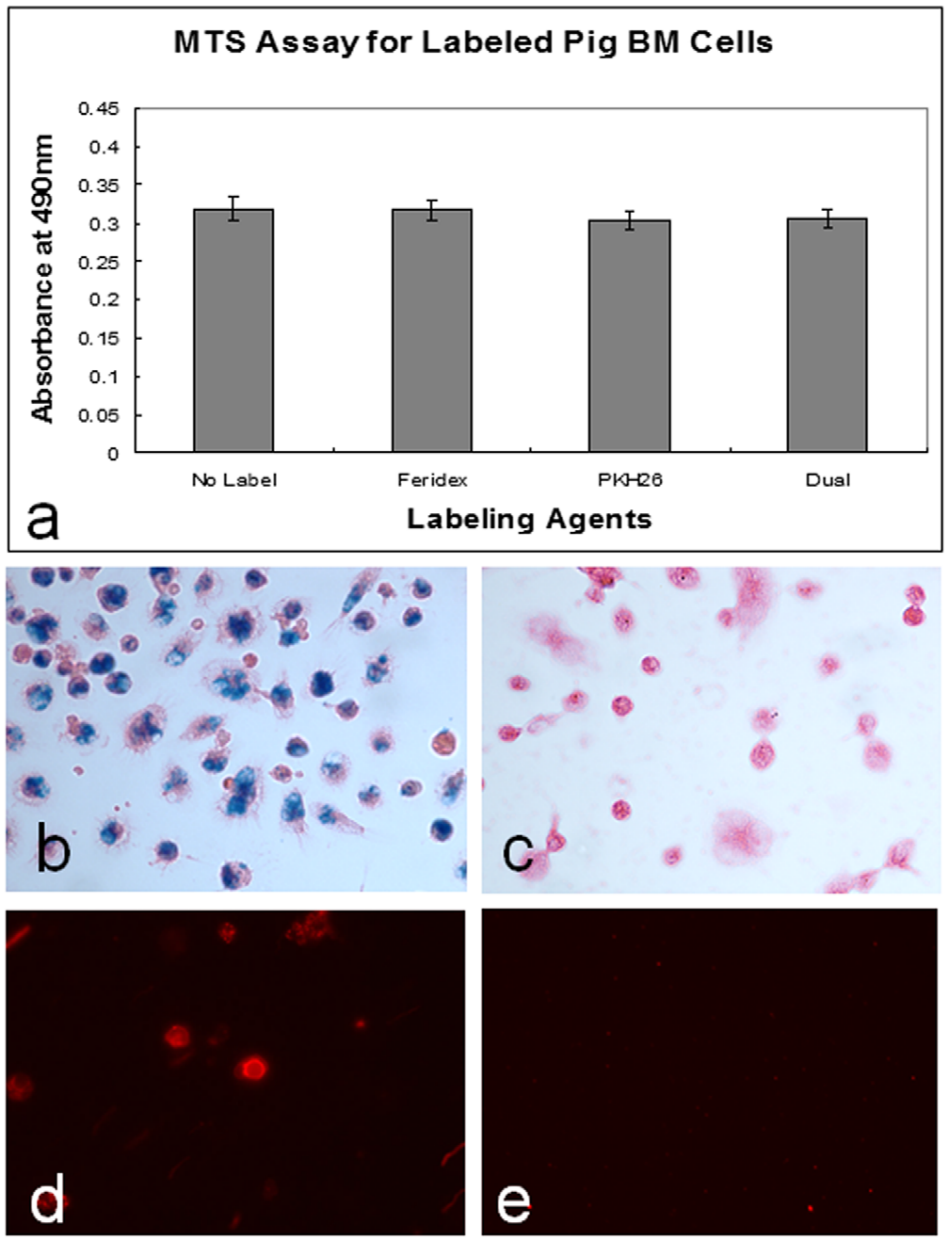
Results of in vitro studies. (a) MTS assay shows the average proliferation efficiencies are almost the same among the different groups of BM-SPCs labeled with Feridex, PKH26, and dual agents (Feridex+PKH26), in comparison with control BM-SPCs without labeling. (b&c) Prussian-blue staining shows Feridex-positive cells as blue color spots, which are not seen in the unlabeled (control) cells (c). (d&e) Fluorescent microscopy demonstrates PKH26-positive BM-SPCs as red/orange-colored fluorescent dots (d), which are not visualized in unlabeled (control) BM-SPCs (e).

For the in vitro evaluation, Trypan blue exclusion showed the viabilities of the labeled cells at 95–98%. Regarding the metabolic rate, MTS assay confirmed no significant difference among the Feridex-labeled cell group (0.319±0.072), PKH26-labeled cell group (0.304±0.059), Feridex/PKH26 dual labeled cell group (0.307±0.062), and the control cell group (with no labeling, 0.319±0.069)(Figure 1). The cell labeling efficacy was achieved at approximately 90% for Feridex and 100% for PKH26. Prussian blue stain displayed Feridex-positive cells as blue-colored spots, and fluorescent microscopy showed PKH26-positive cells as red-colored dots (Figure 1).

**Figure 2:**
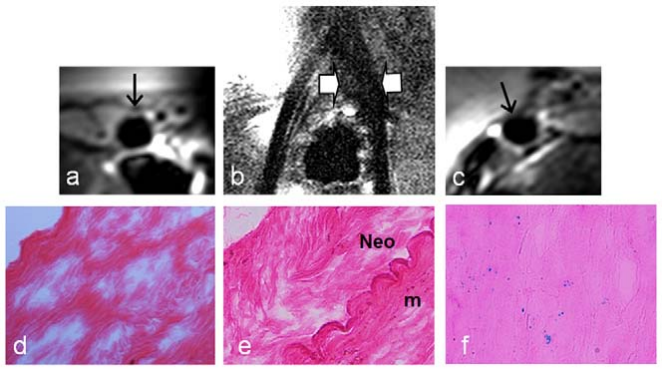
Representative MRI-histology correlation for the auto-transplantation of BM cells in pigs. (b) Near-coronal-view of 3.0T, T2-weighted MRI shows hypo-signal or signal void (between arrows) along the left iliofemoral artery due to the migration of Feridex-labeled BM cells to the injured left iliofemoral artery. (c) Axial-view of intravenous 3.0T MRI shows the disappearance of the anterior wall of the injured left iliofemoral artery (arrow). This is confirmed by histology, showing Feridex-positive cells as blue spots with Prussian blue staining (f). These MRI and histological findings are not seen in the uninjured right iliofemoral artery or injured artery with no cell transplantation (a, b-right iliofemoral artery, d, e). Neo = neointima hyperplasia; m = media.

**Figure 3:**
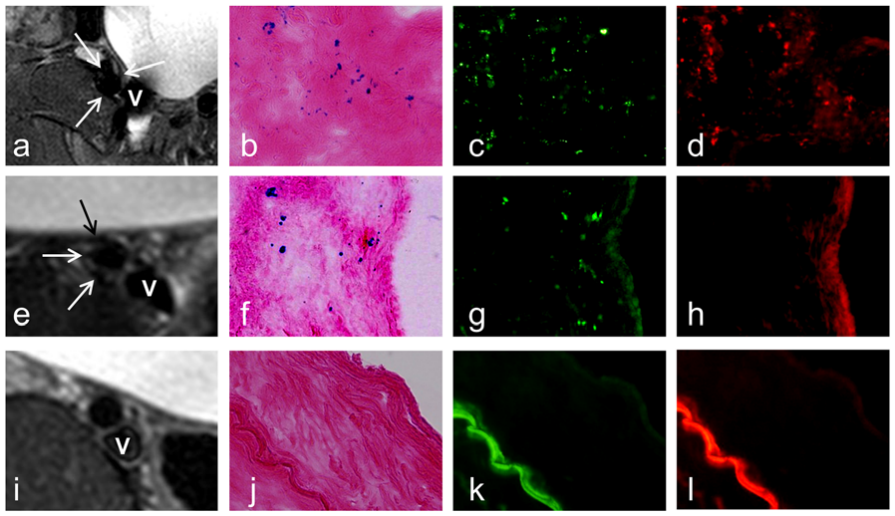
Representative MRI and corresponding histologic findings. (a–d) Animal group 1 with Feridex/PKH26-dual-labeled cell transplantation, showing MR signal void of the iliac artery wall (arrows on a), Prussian blue-positive cells (b), dextran-positive cells (c), and PKH26-positive cells (d), as blue and green as well as red spots or dots, respectively, in the artery wall. (e–h) Animal group 2 with Feridex-only-labeled cell transplantation, demonstrating MR signal void of the iliac artery wall (arrows on e), Prussian blue-positive cells (f), dextran-positive cells (g) and a negative PKH26 stain (h). (i–l) Animal group 3 (control group) with unlabeled cell transplantation or no cell transplantation, showing the intact artery wall on MRI (arrow on i), a negative Prussian blue stain (j), a negative dextran stain (k), and a negative PKH26 stain (l). V = iliac vein.

For the in vivo validation, all 13 pigs survived the experiments through. In the animal group 1with Feridex/PKH26-dual-labeled cell transplantation and the animal group 2 with Feridex-only-labeled cell transplantation, in vivo MRI revealed MR signal voids along the iliofemoral artery walls in 3 of 5 pigs in both animal groups (grade 2 at 60%), whereas similar findings were not seen in the control iliofemoral artery walls (0%) (Figure 2). Histology with H&E staining showed intimal hyperplasia at the injury sites. The intima-to-wall ratio was significantly higher with the intima-injured arteries than with the uninjured arteries (0.20±0.09 vs. 0.05±0.02, *P*<0.01). Fluorescent microscopy detected PKH26-positive cells as red-fluorescent dots in the injured arterial walls in 3 of 5 pigs for animal group 1. Prussian blue staining and immunofluorescence with anti-dextran staining displayed Feridex-positive BM cells as blue-colored spots and green-fluorescent dots, respectively, in 3 of 5 pigs for both animal groups 1 and 2, which were consistent with the MRI and PKH26 findings. These histologic findings were not seen in the control iliofemoral arteries (Figure 3).

## Discussion

It has become clear that, in order to successfully apply cell-based therapy in either experimental or clinical setting, one need to monitor, in vivo, the migration of the transplanted cells. Developments of molecular-cellular imaging techniques enable visualization of cells and their biological activities, such as cell migration [8]. To date, the most commonly-used method for MRI tracking of cell transplantation is to pre-label the cells with various MRI-detectable agents, such as superparamagnetic iron oxide [9], gadolinium [10], fluorine [11], or manganese [12]. Recent efforts have been made using T1 MRI contrast medium for cell labeling, which enabled to not only detect high MRI signal by the labeled cells, but also outline the anatomic structures of the cell-targeted arterial walls [13].

Previous MRI studies using mouse models have demonstrated successfully monitoring of allo-transplantation of Feridex-labeled BM cells homing to the sites of injured arteries or atherosclerotic plaques [6], [7]. However, allo-transplantation of cells requires lethal irradiation of recipients, which has potential impact on vascular cell viability [14]. Most critically, allo-transplantation may cause immunologic rejection and thereby cannot be directly applied in clinical practice on humans. To the best of our knowledge, the current study is the first attempt to validate the feasibility of using clinical 3.0T MRI to track the migration of autotransplanted BM cells to the injured arteries in large animals.

In this study, we first evaluated the possibility of efficiently labeling BM cells with Feridex and/or PKH26, which confirmed that most of labeled cells maintained their normal metabolic activity. We then further validated the feasibility using clinical 3.0T MRI to monitor auto-transplantation of these labeled cells migrated to the injured arteries, which were confirmed by subsequent histologic correlation. We achieved the successful BM cell transplantation in 60% cases, which is similar to the results published by another group using allo-transplantation of BM cells [15]. The reasons for such low engraftment rates are not clear so far, perhaps due to the death of some cells during their handling and transplantation.

In additional to the surface MR coils, we used an MR imaging-guidewire (MRIG). The placement of the MRIG into the iliofemoral veins enabled us to not only avoid the additional mechanical injury to the artery during manipulation of the MRIG passing through the damaged arterial segment, but also achieve satisfactory visualization of the adjacent iliofemoral arteries under high-resolution intravenous MR imaging. In the present study, we also used a multi-slice improved motion-sensitized driven equilibrium sequence (iMSDE). The iMSDE sequence is a black-blood sequence, which can generate higher contrast-to-noise ratio (CNR) and lower lumen signal-to-noise ratio (SNR) in comparison to other commonly-used MR sequences, such as the inflow saturation (IS) and double inversion-recovery (DIR) sequences [16], [17]. The iMSDE sequence can depict a clearer artery contour than other sequences, and thus is capable to generate high resolution 3.0T MRI of the deep-seated arterial walls with sufficient suppression of blood signals. While the signal void created by Feridex-labeled bone marrow cells can obscure the morphology of the arterial lesions in T2-weighted imaging, T1-weighted imaging can be used to image lesion morphology. In our study, the strength of the signal voids was not found to be related to the severities of the artery injuries.

In conclusion, this study demonstrates the capability of using a clinical 3.0T MRI to monitor the auto-transplantation of bone marrow cells that migrate to the injured arteries of near-human-sized animals. Since auto-transplantation of BM cells can avoid immunologic rejection, this technique should be translatable to further clinical application, which may open new avenues to effective management of atherosclerotic cardiovascular disease, the number one killer worldwide, using MRI-integrated regenerative medicine.

## Materials and Methods

**Figure 4:**
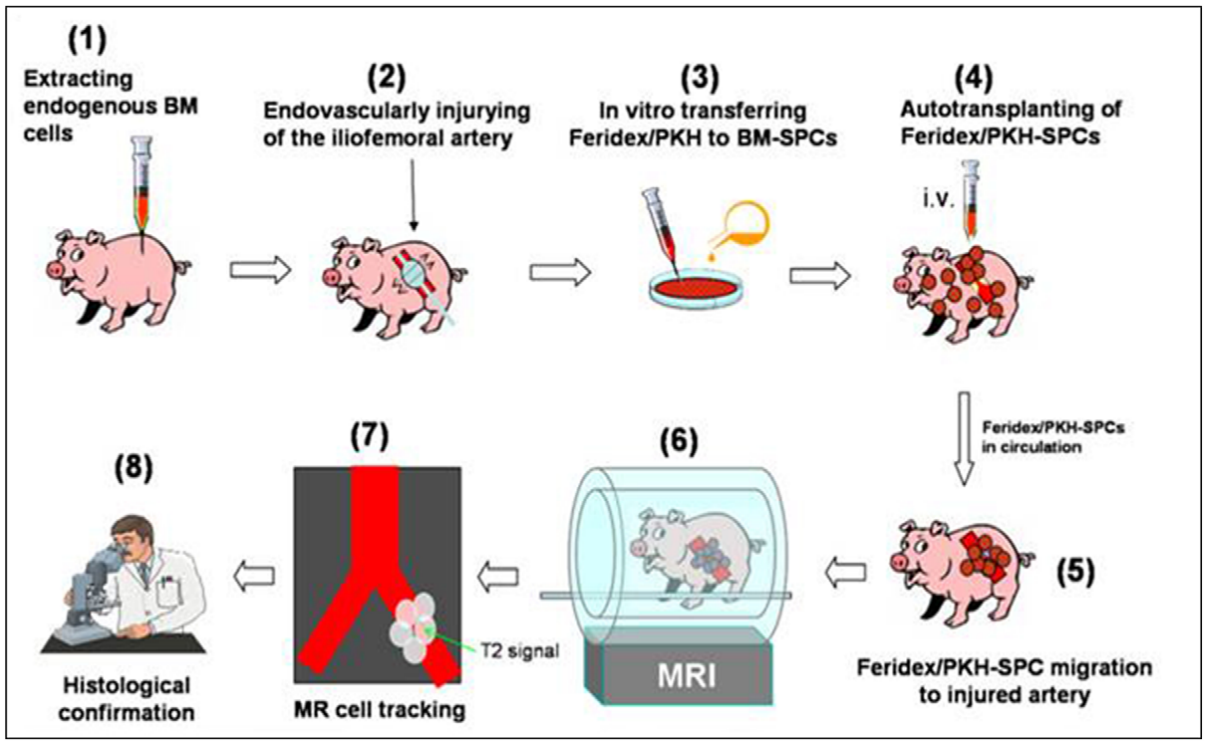
Experimental design and steps. (1) extraction of BM-SPCs from the pig; (2) endovascular intimal injury of the iliofemoral arteries in the same pig; (3) labeling SPCs with Feridex and/or PKH26 in vitro; (4) auto-transplantation of Feridex/PKH26-SPCs back to the same pig; (5) migration of Feridex/PKH26-SPCs to the injured arteries for repair; (6) in vivo 3.0T MR tracking; (7) detection of Feridex-created T2-signals of the Feridex/PKH26-SPCs recruited to the injured arterial segments; and (8) histology confirmation of the success of using MRI to monitor auto-transplantation of Feridex/PKH26-labeled BM-SPCs migrated to the injured arteries for potential cell-based repair.

This study was divided into two components: (i) in vitro evaluating the possibility of labeling BM cells with two agents, a T2-MR contrast agent (Feridex) and/or a fluorescent tissue marker (PKH26); and (ii) in vivo validating the feasibility using clinical 3.0T MRI to monitor the auto-transplantation of these labeled BM cells migrated to the injured iliofemoral arteries of pigs. Figure 4 illustrates the basic experimental design and steps.

### Animals

Animal experiments were designed in compliance with National Institute Health Guideline for the Care and Use of Laboratory Animals, and the animal protocol was approved by the Institutional Animal Care and Use Committee of University of Washington (Approval ID 4120-01). Thirteen domestic pigs (13–50 kg) were divided into three groups: (i) Group 1 (n = 5) with auto-transplantation of BM cells that were co-labeled with Feridex (Bayer HealthCare Pharmaceuticals Inc., Wayne, NJ) and PKH26 (Sigma-Aldrich, St Louis, MO); (ii) Group 2 (n = 5) with auto-transplantation of BM cells labeled with Feridex only; and (iii) Group 3 (n = 3) with unlabeled cell transplantation (one pig) or no cell transplantation (two pigs) to serve as controls.

### In Vitro Evaluation

Under general anesthesia, we extracted approximately 12–20 ml BM from bilateral iliac crests of each pig. We removed red blood cells from BM by using a discontinuous density gradient with Percoll solution (eBioscience, Inc., San Diego, CA), and then collected BM cells at the density interface between 1.067 g/ml and 1.087 g/ml. Then, we labeled BM cells, in vitro, with Feridex and/or PKH26 at 25 µg/ml and poly L-lysine (PLL) at 37.5 ng/ml in QBSP-58 Medium (PeproTech Inc., Rocky Hill, NJ) using the protocol as previously described [18], [19].

The viabilities of the labeled cells were evaluated using Trypan blue exclusion with cell counting. The cell metabolic activity was assessed with 3-(4,5-dimethylthiazol-2-yl) -5-(3-carboxymethoxyphenyl)-2-(4-sulfophenyl)-2H-tetrazolium (MTS) using CellTiter 96 AQ_ueous_ one solution cell proliferation assay (Promega, Madison, WI) [20]. Feridex labeling efficacy was determined by counting the labeled cells [21] after Prussian blue staining, while PKH26 labeling efficacy determined by counting labeled cells under a fluorescent microscope. We randomly selected five microscope fields to count the labeled cells from six cell groups, and then calculated the average cell labeling efficacies.

### In Vivo Validation

#### Creation of animal models with arterial injuries

After sedation, each pig underwent general anesthesia with inhalation of 2% isoflurane mixed with 1 L/min oxygen. Through a surgical cut-down or percutaneous access, a Fogarty balloon (Edwards Lifesciences , Irvine, CA) or a cutting balloon (Boston Scientific, Natick, MA) was positioned into the iliac artery 2-cm below the bifurcation, where intima was injured by pulling the over-inflated Fogarty balloon or over-inflating the cutting balloon under ultrasound imaging guidance. Each pig was created two injury sites in either one or two iliofemoral arteries. Then, the accessed femoral artery segment was ligated using sutures to achieve hemostasis at the access site. One day after the endovascular intimal injury, 2.6×10^7^ labeled or unlabeled BM cells suspended in 10-mL phosphate-buffered-saline were autotransplanted, via an ear vein injection, back to the same pig from which the BM cells were extracted.

#### MRI

Three weeks post-cell transplantation, each pig was imaged using a 3.0T MR scanner (Achieva, Philips Healthcare, Best, Netherlands) to detect the BM cells migrated to the injured iliofemoral arterial segments. MRI was performed using a surface coil (n = 13) placed at the anterior and posterior pelvic, and a custom made 0.032-inch MR imaging-guidewire (MRIG) (n = 12). The custom-made MRIG was manufactured from a 0.032-inch nitinol coaxial cable with an extension of 3.2-cm inner conductor from the outer conductor. The tip of the MRIG was refined with a solenoid spring, which facilitated the smooth advance of the MRIG into vessels and avoided the incidence of vessel injury during the endovascular manipulation. The proximal end of the MRIG was connected to 3.0T MR through a matching/tuning/decoupling circuit [22]. For MRI using MRIG, the MRIG was placed, via a femoral vein access, into the iliofemoral vein, where intravenous MRI of the adjacent iliofemoral artery was archived. MR parameters included an improved motion sensitized driven equilibrium (iMSDE) sequence for coronal and axial T2-weighted imaging of the target vessels at 4500–5000 ms TR, 40 ms TE, 90°flip angle, 10 echo train length, 100×100 mm FOV, 144×140 matrix, 3 mm slice thickness, and 8 NEX. For each of iliofemoral arteries, a total of 15–20 slices were achieved.

#### Histologic correlation

After achieving satisfactory MR images, the pigs were euthanized by intravenous injections of pentobarbital sodium (100 mg/kg). The iliofemoral arteries were harvested and cryosectioned at 5-µm slice thickness through. We then stained the 15–20 slices with (a) hematoxylin and eosin (H&E) staining for histologically grading the arterial injuries; (b) Prussian blue staining for detecting Feridex-positive cells; (c) immunofluorescent staining for detecting dextran shells of Feridex; and (d) fluorescent microscopy examination for detecting PKH26-positive cells in the target vessel walls. Since this study mainly focused on development of the new MR technique, we did not attempt to examine the downstream phenotypes of the migrated BM cells.

#### Data analysis

MR images were analyzed with Philips DICOM viewer software. Manifestations of the arterial walls were divided into 2 grades: Grade 1 with intact arterial walls as a bright ring, and Grade 2 with incomplete bright ring or missed portion of arterial walls due to migrated Feridex/BM cell-created MR signal void. Of H&E staining, the thickness of the intimal hyperplasia and the artery wall was measured and the intima-to-wall ratio (IWR_injure_) was calculated and compared to IWR_control_.

### Statistics

Statistic analysis was performed using SPSS software. ANOVA was used to compare the cell proliferation efficiencies between labeled and controlled BM cells. Non-parametric Mann-Whitney U test was used to compare the IWRs between injured and uninjured artery walls. Data was expressed as mean ± SD. A probability level of less than 0.05 was considered statistically significant.
